# Whole-Genome Sequence Characterization of a Beak and Feather Disease Virus in a Wild Regent Parrot (*Polytelis anthopeplus monarchoides*)

**DOI:** 10.1128/genomeA.01243-13

**Published:** 2014-01-30

**Authors:** Subir Sarker, Jade K. Forwood, Seyed A. Ghorashi, David McLelland, Andrew Peters, Shane R. Raidal

**Affiliations:** a School of Animal and Veterinary Sciences, Charles Sturt University, Wagga Wagga, New South Wales, Australia; b School of Biomedical Sciences, Charles Sturt University, Wagga Wagga, New South Wales, Australia; c Graham Centre for Agricultural Innovation, Wagga Wagga, New South Wales, Australia; d Zoos South Australia, Adelaide, South Australia, Australia

## Abstract

The whole-genome sequence of beak and feather disease virus (BFDV) from a wild Australian regent parrot (*Polytelis anthopeplus monarchoides*) was characterized. The genome consists of 1,993 bp and has a typical stem-loop structure between open reading frame 1 (ORF1) and ORF2. This is the first evidence of BFDV infection as well as the complete genome sequence for this host species, globally.

## GENOME ANNOUNCEMENT

Beak and feather disease virus (BFDV) is one of the smallest and simplest pathogens, in terms of both genome and particle size, of any vertebrate, and it causes psittacine beak and feather disease (PBFD). It belongs to the family *Circoviridae* (1–3), with the circular single-stranded DNA (ssDNA) genome of BFDV surrounded by a nonenveloped icosahedral capsid 19 to 22 nm in diameter, and it must use host cell machinery to replicate (4, 5). The emergence of the disease in critically endangered species has been recently highlighted as a key threat to the orange-bellied parrot (6). It is estimated that no more than 2,900 adult regent parrots (*Polytelis anthopeplus monarchoides*) exist across the range of the Murray-Darling river catchment of southeastern Australia; as a result, the regent parrot is also currently listed as vulnerable under the Australian Government *Environment Protection and Biodiversity Conservation Act 1999* (EPBC Act) and endangered under the NSW *Threatened Species Conservation Act 1995* (7). PBFD presents with chronic symmetrical feather loss, as well as beak and claw deformities eventually leading to death or acutely infected juvenile birds, causing sudden death even in the absence of clinical signs (5, 8–10). Here, BFDV is reported for the first time in a wild regent parrot in Australia.

Dried blood spots collected from a regent parrot (sample ID, 13-1683; year of sampling, 2013; global positioning system [GPS] location, 34.100°S 140.4440°E) was used as a source of genomic DNA, and extraction of DNA was performed using established protocols (11–13). The whole-genome sequence was amplified using the primers and PCR conditions described by Sarker et al. (14, 15). Briefly, the optimized reaction mixture contained 3 µl extracted genomic DNA, 2.5 µl of 10× High Fidelity PCR buffer (Invitrogen), 1 µl of 25 µM each primer, 1 µl of 50 mM MgSO_4_, 4 µl of 1.25 mM dinucleoside triphosphates (dNTPs), 1 U platinum *Taq* DNA Polymerase High Fidelity (Invitrogen), and dH_2_O added for a final volume of 25 µl. The optimized PCR conditions were 95°C for 3 min, followed by 40 cycles of 95°C for 30 s, 57°C for 45 s, and 68°C for 2 min, and finally 68°C for 5 min. Amplified PCR products were TA cloned into pGEM-T vector (Promega, USA) and sequenced at the Australian Genome Research Facility (AGRF), Ltd. (Sydney, Australia). The sequenced contigs were assembled, and the entire BFDV genome was constructed using Geneious software (version 6.1.6).

The complete genome consists of 1,993 bp, with a G+C content of 53.5%. The genome has the same basic structure as other BFDV genomes, which includes two major open reading frames (ORFs), ORF1 on the virion strand, encoding a replication-associated protein (Rep), and ORF2, encoding the capsid protein (Cap). The overall nucleotide (nt) identity of this new isolate compared with the BFDV genome available on GenBank (16) ranges from >82 to 95%. Consequently, based on BLASTn (17) and BLASTp (18), ORF2 is more diverse than ORF1. The nucleotide identity of ORF2 of the regent parrot BFDV genome varies from 88 to 95% compared with other BFDV genomes, while the identity of the amino acid sequence ranges from 77 to 96%.

This study highlights the evidence of BFDV infection in an endangered regent parrot, which may provide novel insights into the management and recovery of this host species.

### Nucleotide sequence accession number.

This complete genome of BFDV has been deposited at DDBJ/EMBL/GenBank under the accession no. KF850537. The version described in this paper is the first version.
